# The Chemical Biology of Human Metallo-β-Lactamase Fold Proteins

**DOI:** 10.1016/j.tibs.2015.12.007

**Published:** 2016-04

**Authors:** Ilaria Pettinati, Jürgen Brem, Sook Y. Lee, Peter J. McHugh, Christopher J. Schofield

**Affiliations:** 1 Department of Chemistry, University of Oxford, 12 Mansfield Road, Oxford, OX1 3TA, UK; 2 Weatherall Institute of Molecular Medicine, University of Oxford, John Radcliffe Hospital, Oxford, OX3 9DS, UK

**Keywords:** β-lactam antibiotic and cancer drug resistance, metallo β-lactamase fold protein, DNA repair, RNA processing, hydrogen sulphide metabolism, nuclease

## Abstract

The αββα metallo β-lactamase (MBL) fold (MBLf) was first observed in bacterial enzymes that catalyze the hydrolysis of almost all β-lactam antibiotics, but is now known to be widely distributed. The MBL core protein fold is present in human enzymes with diverse biological roles, including cell detoxification pathways and enabling resistance to clinically important anticancer medicines. Human (h)MBLf enzymes can bind metals, including zinc and iron ions, and catalyze a range of chemically interesting reactions, including both redox (e.g., ETHE1) and hydrolytic processes (e.g., Glyoxalase II, SNM1 nucleases, and CPSF73). With a view to promoting basic research on MBLf enzymes and their medicinal targeting, here we summarize current knowledge of the mechanisms and roles of these important molecules.

## Trends

MBLs are mono- or di-zinc ion-dependent hydrolases that enable bacterial resistance to almost all β-lactam antibiotics.

The αββα MBL core fold is widely distributed and supports a range of catalytic activities, including redox reactions.

hMBL proteins are a small family of approximately 18 zinc- and iron-dependent proteins with roles in metabolism and/or detoxification and nucleic acid modification.

In a notable parallel with the role of bacterial MBLs in antibiotic resistance, some hMBLf enzymes enable resistance to chemotherapy drugs, such as cisplatin and mitomycin C.

## Overview of the MBL Superfamily

The β-lactam antibiotics were discovered over 75 years ago, yet remain among the most important medicines. One of the most important bacterial resistance mechanisms to β-lactam antibiotics involves their hydrolysis, as catalyzed by two mechanistically distinct families: the nucleophilic serine β-lactamases and the zinc ion-dependent MBLs (Class B β-lactamases) 1, 2.

In recent years, MBL-mediated antibiotic resistance, in particular to carbapenems (often antibiotics of last resort), has become widely disseminated. MBLs belonging to subclass B1, such as the New Delhi metallo-β-lactamase 1 (NDM-1), are of widespread clinical significance [3]. Following the successful development of serine β-lactamase inhibitors (Box 1), MBL inhibitors are being developed [4], but none have yet been developed for clinical use.

The determination of crystal structures for prokaryotic β-lactam hydrolyzing MBLs (class B β-lactamases) led to the discovery of a novel αββα protein fold and the finding that the MBLf is widely distributed in biology, including in humans, where it supports diverse roles 5, 6, 7. To date, the MBL superfamily includes >34 000 predicted members of diverse function and metal utilization (see 7, 8, 9 for recent detailed reviews). Most of the characterized and predicted MBLf enzymes are hydrolases acting on nucleic acids and small molecules (including β-lactam antibiotics), but they can also catalyze different types of reaction, including in redox chemistry, such as during detoxification of hydrogen sulfide [10]. In a notable parallel with the role of bacterial MBLs in antibiotic resistance, some hMBLf enzymes (e.g., the DNA crosslink repair enzymes SNM1A and B) enable resistance to anticancer drugs of major clinical relevance, including mitomycin C and cisplatin [11]. Work on hMBLf enzymes is of fundamental enzymology interest because of the range of reactions catalyzed by a single protein fold 7, 8, 9; it is also of medicinal relevance because it may help both enable development of selective inhibitors for the prokaryotic β-lactam-hydrolyzing MBLs and in the development of drugs to overcome resistance to chemotherapeutic agents. In this review, we summarize current knowledge of hMBLf proteins, with a view to promoting basic and medicinal research on these important molecules.

## Overview of hMBLf Enzymes

**Figure 1:**
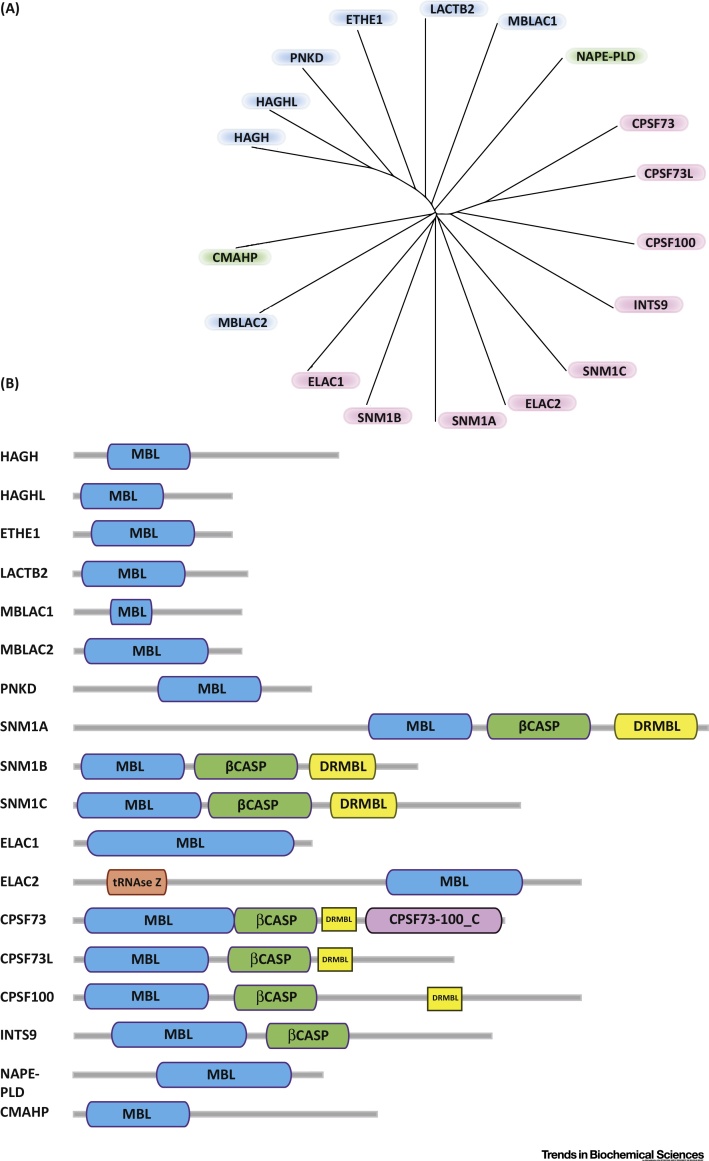
Phylogeny and Domain Architecture of Human Metallo β-Lactamase Fold (hMBLf) Enzymes. (A) Phylogenetic tree representation of the hMBLf superfamily generated using the Clustal Omega multisequence alignment tool [111]. (B) Domain architecture of hMBLf enzymes. Note that the DRMBL motif, if present, is shown as a small yellow block [112]. Abbreviations: CMAHP, CMP-*N*-acetylneuraminic acid hydroxylase pseudogene; CPSF100, cleavage and polyadenylation specificity factor 100; CPSF73, cleavage and polyadenylation specificity factor 73; CPSF73L, cleavage and polyadenylation specificity factor 73 like; DCR1A (SNM1A), DNA crosslink repair 1A; DCR1B (SNM1B/Apollo), DNA crosslink repair 1B; DCR1C (SNM1C/Artemis), DNA crosslink repair 1C; DRMBL, DNA repair metallo β lactamase; ELAC1 and 2, zinc phosphodiesterase ELAC protein 1 and 2; ETHE1, ethylmalonic encephalopathy protein 1; HAGH, hydroxyacylglutathione hydrolase or Glyoxalase II; HAGHL, hydroxyacylglutathione hydrolase or Glyoxalase II like; INTS9, integrator complex subunit 9; LACTB2, β-lactamase-like protein 2; MBLAC1 and 2, MBL domain-containing protein 1 and 2; NAPE-PLD, *N*-acyl-phosphatidylethanolamine-hydrolyzing Phospholipase D; PNKD, paroxysmal nonkinesiogenic dyskinesia protein; RNAse Z, ribonuclease Z; β-CASP, CPSF-Artemis-SNM1-Pso2.

Structurally informed sequence analyses reveal (at least) 18 hMBLf proteins, all of which contain some of the conserved active site ‘motifs’ present in β-lactam antibiotic-hydrolyzing prokaryotic MBLs. In hMBLf enzymes, there are five identified active site motifs: (i) H_84_; (ii) H_116_XH_118_XD_120_H_121_; (iii) H_196_; (iv) D_221_; and (v) H_263_, with the first three being best conserved 7, 8. Human motif (i) (H_84_) is not present in the β-lactam antibiotic-hydrolyzing prokaryotic MBLs and motif (iv) (D_221_) replaces the prokaryotic C_221_ motif. Phylogenetic analyses (Figure 1) cluster hMBLf proteins into three groups or subfamilies. Group 1 comprises glyoxalase II family-related enzymes, Group 2 comprises enzymes involved in nucleic acid modifications, including some linked to anticancer drug resistance, and Group 3 comprises hMBLf proteins with more diverse functions. For a general overview of hMBLf enzyme function, localization, and metal utilization, see Table 1. Here, we describe work on these subfamilies, beginning with the glyoxalase II-related enzymes, some of which act on small molecules in a manner related to β-lactam chemistry.

## Group 1 hMBLf Enzymes: The Glyoxalase II Subfamily

The Glyoxalase II subfamily of hMBLf enzymes comprises seven members: hydroxyacylglutathione hydrolase (HAGH) or Glyoxalase II itself; hydroxyacylglutathione hydrolase-like protein (HAGHL); ethylmalonic encephalopathy protein 1 (ETHE1); MBL domain-containing protein 1 (MBLAC1); MBL domain-containing protein 2 (MBLAC2); beta-lactamase-like protein 2 (LACTB2); and paroxysmal nonkinesiogenic dyskinesia protein (PNKD) (Table 1). Of the Glyoxalase II hMBLf proteins, biochemical functions have been assigned for two (HAGH and ETHE1).

### HAGH

HAGH catalyzes a key step in the detoxification of reactive and toxic 2-oxoaldehydes, including methylglyoxal [12]. 2-Oxoaldehydes react with DNA and proteins, resulting in aberrant postoligomerization modifications, which are associated with aging by causing DNA instability and alteration of protein function [13].

**Figure 2:**
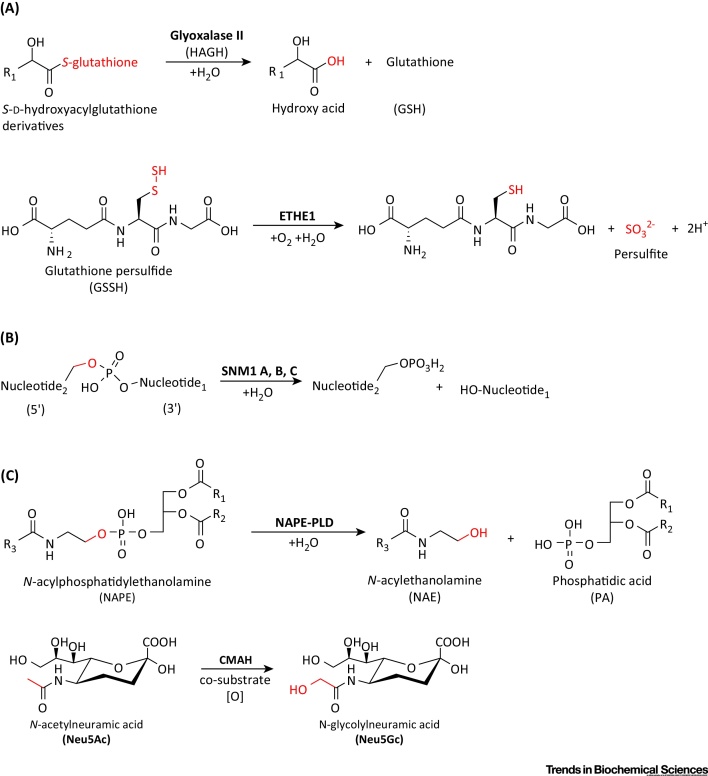
Known Reactions Catalyzed by Human Metallo β-Lactamase Fold (hMBLf) Enzymes (and Cytidine Monophospho-*N*-Acetylneuraminic Acid Hydroxylase). (A) Group 1, glyoxalase II, and human ethylmalonic encephalopathy protein 1 (hETHE1); (B) Group 2: DNA crosslink repair 1 (DCR1 or SNM1A, B, and C) enzymes; (C) Group 3: *N*-acyl-phosphatidylethanolamine-hydrolyzing phospholipase D (NAPE-PLD) and an enzyme present in most if not all mammals except humans, cytidine monophospho-*N*-acetylneuraminic acid hydroxylase (CMAH). Substrate atoms directly involved in enzyme catalysis are in red. Note that not all hMBLs have assigned functions.

The well-characterized glyoxalase system converts the toxic 2-oxoaldehydes to less-reactive metabolites [13] and comprises two key enzymes: Glyoxalase I and HAGH [13]. Glyoxalase I, which is not an MBLf protein, catalyzes the first step; that is isomerization of a nonenzymatically formed hemithioacetal adduct of the ubiquitous reducing agent glutathione and the toxic 2-oxoaldehyde, to give a (*S*)-2-hydroxyacylglutathione thioester [14]. HAGH then catalyzes hydrolysis of the thioester to give a 2-hydroxycarboxylate product (e.g., lactate from methylglyoxal) and regenerate glutathione (Figure 2A) [13].

**Figure 3:**
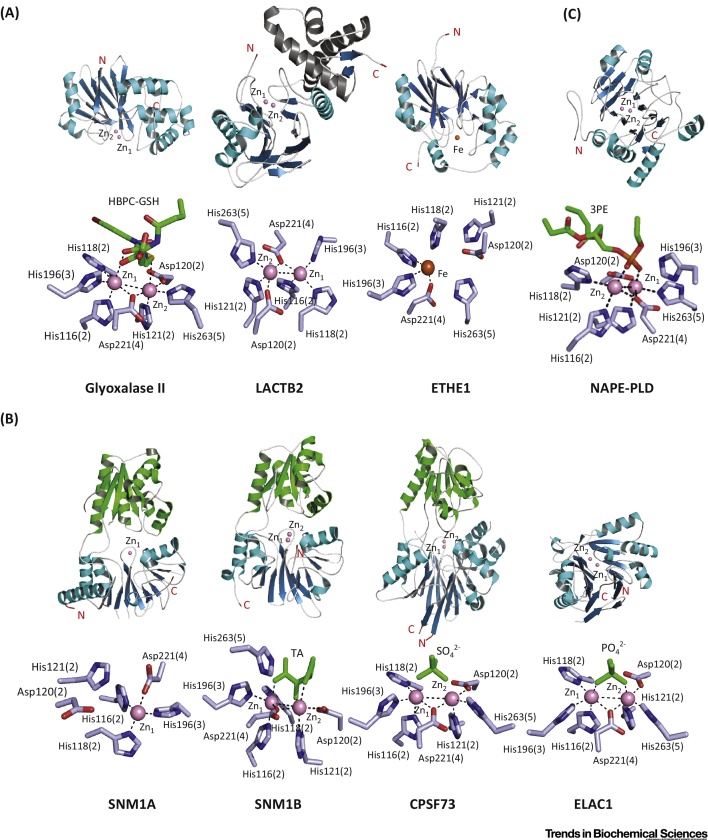
Overall Folds and Active Sites of Human Metallo β-Lactamase Fold (hMBLf) Enzymes. (A) Glyoxalase II family hMBLs: glyoxalase II [Protein Data Bank (PDB) ID: 1QH5], LACTB2 (PDB ID: 4AD9), ETHE1 (PDB ID: 4CHL). (B) DNA/RNA-interacting hMBLs: SNM1A (PDB ID: 4B87), SNM1B (PDB ID: 5AHO), CPSF73 (PDB ID: 2I7T) and ELAC1 (PDB ID: 3ZWF). (C) Group 3 hMBLs: NAPE-PLD (PDB ID: 4QN9). The crystallographic studies reveal how the conserved MBLf supports different types of reaction. Given the varied reaction types catalyzed by MBLf enzymes, the metal-coordination chemistry for the different enzymes appears to be similar; however, differences are such that the development of selective inhibitors should be possible. Note the conservation of some residues in binding the ‘second’ zinc in enzymes that only bind one metal (e.g., ETHE1) and that work on the accurate definition of metal use by nonheme metallo-proteins is limited by availability of methods for quantifying metal binding by proteins at endogenous levels. It should also be noted that the use of different metals can alter the catalytic profile of the same MBLf enzyme [113]. The MBL domain is in blue (β-sheets) and cyan (α-helices); β-CPSF-Artemis-SNM1-Pso2 (β-CASP) domains are in green. *N-* and *C*-terminal residues are in red. Zinc and iron ions are in pink and orange, respectively. Sulfate and phosphate ions are in green. Substrates and inhibitors, if present, are in green. The standard BBL MBL numbering system is used [103], with the active site motif number in parentheses (see main text). Abbreviations: 3PE, 1,2-diacyl-sn-glycero-3-phosphoethanolamine [note that, in (C), part of the 3PE molecule is omitted to show active site interactions]; HBPC-GSH, *S*-(*N*-hydroxy-*N*-bromophenylcarbamoyl) glutathione; TA, L(+)-tartaric acid. Note that there are few high-quality full-length (i.e., including noncatalytic domains) enzyme–substrate and/or product structures reported for nonclassical MBLs; product complex structures are reported for classical MBLs [114].

**Figure 4:**
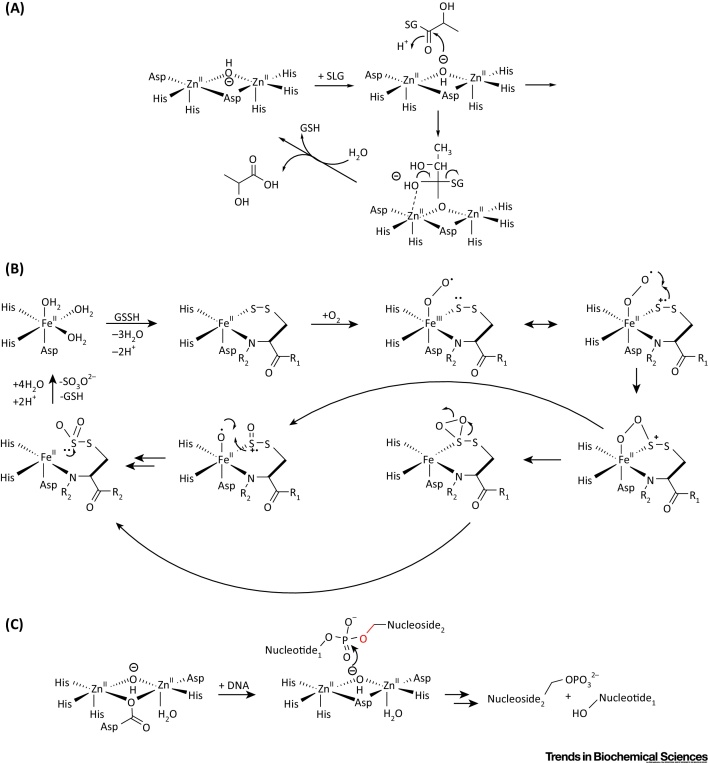
Outline Mechanism for Reactions Catalyzed by Human Metallo β-Lactamase Fold (hMBLf) Enzymes. (A) Outline of the Glyoxalase II mechanism, showing the proposed tetrahedral intermediate [16]. (B) Proposed ethylmalonic encephalopathy protein 1 (ETHE1) mechanism; initial iron complexation of GSSH thiol and GSSH cysteinyl-glycine amide nitrogen to the iron (II) is followed by binding of O_2_, then oxidation of the ligated disulfide 10, 30. (C) Possible outline mechanism for the SNM1-type nucleases.

Following the determination of a crystal structure for HAGH [Protein Data Bank (PDB) ID:1XM8] from *Arabidopsis thaliana*, which revealed a di-zinc binding site [15], human HAGH structures have been reported, including in complex with glutathione and the substrate analog *S*-(*N*-hydroxy-*N*-bromophenylcarbamoyl)glutathione (PDB ID: 1QH3/5); this is one of the few MBL structures providing detailed insights into substrate binding [16] (Figure 3A). As with other MBLf superfamily members, HAGH can use metal ions other than zinc, including iron and manganese ions [17]. Of the characterized hMBLf proteins, the HAGH reaction is perhaps the most closely related to that of classical MBLs. The proposed HAGH mechanism involves nucleophilic attack of water onto the substrate carbonyl to give a negatively charged tetrahedral intermediate that is stabilized by a zinc ion, followed by C–S bond cleavage (Figure 4A). In this regard, it is notable that, in solution, the proposed rate-limiting step in HAGH catalysis and, at least in some cases of β-lactam hydrolysis, is breakdown of the tetrahedral intermediate, whereas in acyclic amide hydrolysis, it is (normally) formation of the tetrahedral intermediate. Interestingly, some β-lactam antibiotics hydrolyzing MBLs can catalyze thioester hydrolysis in a mechanism related to that of HAGH [9], and thiols are an established class of MBL inhibitors 18, 19.

### ETHE1

ETHE1 has a key role in the mitochondrial metabolism of the toxic (and smelly) small molecule H_2_S. In mitochondria, ETHE1 catalyzes the oxygen-dependent conversion of the persulfide (GSSH) formed from glutathione and H_2_S to give glutathione and persulfite [10] (Figure 2A). Mutations in the *ETHE1* gene correlate with the autosomal recessive metabolic disease ethylmalonic encephalopathy (EE), which leads to death in infancy 20, 21, 22. To date, 16 clinically observed mutations have been identified in *ETHE1*, at least some of which are likely inactivating. Substitutions at the conserved Arg163, located on a loop in close proximity to the active site, are proposed to cause reduced protein stability and alteration of the redox potential of the iron center [23]. However, some missense mutations occurring at highly conserved residues lead to normal or reduced levels of active ETHE1 10, 20, 24, 25.

ETHE1 is overexpressed in hepatocellular carcinoma cells and is proposed to abrogate apoptosis mediated by the transcription factor p53, thereby enhancing cancer progression 26, 27. Two possible mechanisms have been proposed for this process. ETHE1 is proposed to act as a nuclear-cytoplasmic shuttling protein that binds the nuclear factor kappa-light-chain-enhancer of activated B cells (NF-κB) protein RelA/p65 in the nucleus and promotes its transport to the cytoplasm. Given that the nuclear localization of NF-κB is essential in p53-mediated apoptosis, the ETHE1-dependent sequestration of RelA/p65 in the cytoplasm results in failure of p53-mediated cell death [26]. Alternatively, it is proposed that ETHE1 may reduce p53-regulated transcription of proapoptotic genes by interacting with histone deacetylase 1 (HDAC1) and thereby promotes p53 deacetylation [27]. Given the roles of ETHE1 in thiol metabolism and the role of metal ions in both HDAC and hMBLf catalysis, it is notable that HDAC inhibitors are often metal chelators, including appropriately functionalized thiols and hydroxamic acids. These classes of HDAC inhibitor are known classes of ‘classical’ MBL inhibitor [28]. It is possible that the biological activities of such inhibitors, including effects on cell proliferation, arise from inhibition of hMBLf proteins (including ETHE1) in addition to HDACs.

From a mechanistic perspective, ETHE1 is remarkable. Biochemical and structural studies reveal that it binds a single Fe (III) ion, instead of Zn (II), into its active site 10, 29. The human ETHE1 structure (PDB ID: 4CHL) [30] is closely related to that of ETHE1 from *A. thaliana* and reveals a dimer with a single iron ion at each active site [30] (Figure 3A). Interestingly, in both the *A. thaliana* and human ETHE1 structures, an oxidized cysteine residue is present close to the active site, although the role of this modification in unknown 29, 30. In a manner closely reminiscent of the nonheme iron oxygenases of the 2-oxoglutarate oxygenase and other superfamilies, ETHE1 uses a 2-His-carboxylate site motif to complex its nonheme iron. The proposed ETHE1 mechanism is related, at least in some aspects, to that of the key enzyme in penicillin biosynthesis [i.e., isopenicillin N synthase (IPNS) [31]], and involves initial complexation of the GSSH thiol and cysteinyl-glycine amide nitrogen to the iron followed by binding of O_2_ and oxidation of ligated sulfur or disulfide 10, 30 (Figure 4B). Further work is required to characterize the proposed intermediates in ETHE1 catalysis.

### PNKD

PNKD exists in four different isoforms (due to alternate splicing), of which isoforms 1, 3, and 4 contain an MBLf domain, whereas isoform 2 does not [32]. The biochemical role and mechanism of PNKDs are unknown. The PNKD MBLf domain has high sequence similarity with HAGH, and PNKD is proposed as a Glyoxalase II-type enzyme [33]. Mutations of the *PNKD* gene correlate with paroxysmal nonkinesigenic dyskinesia or Dystonia type 8 (DYT8), which is characterized by dystonia, chorea, and athetosis (i.e., involuntary muscle contractions) 33, 34, 35. The observation of dysfunction in dopamine signaling (i.e., increased dopamine turnover) in the basal ganglia of PNKD-1-knockout mice suggests that the dopamine pathway is involved in paroxysmal nonkinesigenic dyskinesia 33, 36. In support of this, mutant PNKD-expressing mice display alterations in exocytosis, suggesting that PNKD is involved in modulation of neurotransmitter release at the nigrostriatal dopaminergic terminals [37]. PNKD is present in muscle tissues including skeletal muscle and myocardial myofibrils, where it interacts with sarcomeric proteins, indicating a role for it as a myofibrillogenesis regulator 38, 39, 40. PNKD2 stimulates fiber formation and, indirectly, activates the focal adhesion kinase/(Ser/Thr) kinase (FAK/Akt) signaling pathway to promote cell migration and proliferation [41]. PNKD2 is overexpressed in some cancer cells (e.g., ovarian) and is proposed to be involved in tumor cell proliferation, migration, and adhesion [42]. Although the biochemical roles of PNKD have not yet been identified, collectively these studies suggest that PNKD is involved in multiple cellular pathways, and its malfunction has pleiotropic consequences.

The other Glyoxalase II hMBLf subfamily proteins are of unassigned function. Sequence analyses suggest that MBLAC1/2, HAGHL. and LACTB2 bind two metal ions [as supported by crystallographic analyses for LACTB2 (PDB ID: 4AD9)] and may be hydrolases (Figure 3A).

### Group 1 hMBLf Enzymes: Summary

Of the seven members of the glyoxalase II subfamily, biochemical functions have been assigned only for two (HAGH and ETHE1). However, the established and apparently physiologically essential roles of HAGH and ETHE1 in detoxification and pleiotropic aspects of biology, along with the disease-associated links of PNKD, imply that the Glyoxalase II subfamily of hMBLf enzymes likely has more central roles in metabolism than was previously perceived.

## Group 2 hMBLf Enzymes: The DNA/RNA Interacting hMBLf Subfamily

The DNA/RNA interacting hMBLf subfamily is the largest hMBLf subfamily, comprising nine proteins that are mostly nucleases, and are involved in DNA repair and somatic recombination in the immune system (SNM1 A, B, and C), and RNA processing (ELAC1-2 and the CPSFs) [43] (Figure 2B). Six of them (SNM1 A, B, C, CPSF73, CPSF73-like, and CPSF100) also contain a discrete β-CPSF-Artemis-SNM1-Pso2 (β-CASP) domain, which along with the MBLf domain is involved in nucleic acid binding 44, 45. The SNM1 enzymes (SNM1 A, B, and C) are orthologs of the budding yeast enzyme Pso2, and have been found to have important roles in eukaryotic DNA damage repair 46, 47. In an interesting parallel with the role of prokaryotic MBLs in resistance to β-lactam antibiotics, the SNM1 nucleases are involved in resistance to antitumor drugs.

### DNA Crosslink Repair 1A and 1B Enzymes (DCR1A and 1B or SNM1A and 1B)

DNA crosslink repair 1A (SNM1A or DCR1A) and DNA crosslink repair 1B (SNM1B, DCR1B, or Apollo) are 5́-3́ exonucleases that are activated on DNA damage. SNM1A/B are important in interstrand crosslink (ICL) repair; in particular, SNM1A has a striking ability to resect past site-specific crosslinks *in vitro* [48]. Cells depleted in SNM1A/B show increases in sensitivity to ICL-inducing agents, such as cisplatin and mitomycin C [49]. Mutations of the SNM1A and SNM1B MBLf domains cause loss of nuclease activity, revealing the crucial role of the MBLf domain in repair 49, 50. Cells depleted of SNM1A accumulate double-strand (ds) breaks (DSB) as a consequence of replication fork breakage. These observations indicate a role for SNM1A in the predominant, replication-coupled, ICL repair pathway. In support of this, in mammalian cells, SNM1A promotes G_1_ cell cycle checkpoint arrest after exposure to ionizing radiation 51, 52.

SNM1B has an established role in telomere maintenance via interaction with the telomere-associated factor (TRF2) [50]. SNM1B is involved in the generation of 3′-overhang structures at telomeres, protecting them from deleterious repair by nonhomologous end-joining (NHEJ) [53]. Similarly to SNM1A, depletion of SNM1B sensitizes cells to ICLs as well as to other genotoxins, such as ionizing radiation and cisplatin 54, 55, 56.

### DNA Crosslink Repair 1C (DCR1C, SNM1C, or Artemis)

SNM1C has a key role in one of the major pathways for repair of DSB in mammalian cells: the NHEJ pathway [47]. SNM1C is also involved in antibody and T cell diversification as a consequence of its role in V(D)J recombination, a specialized somatic recombination process that is essential for adaptive immunity [57]. SNM1C is a 5′-3′ exonuclease acting on both single-strand (ss) and ds DNA and, uniquely within the DNAase MBLf family, is also an endonuclease that acts on DNA hairpin structures generated during V(D)J recombination and on DNA overhangs during NHEJ repair of ds breaks. The endonuclease activity of SNM1C is promoted by its phosphorylation and complexation with DNA-dependent protein kinases 58, 59. Mutations of the SNM1C MBLf domain correlate with radiosensitive severe combined immunodeficiency syndrome (RS-SCID), likely due to impaired V(D)J recombination 57, 59 and defects in repair of ionizing-radiation-induced ds breaks. The C-terminal region of SNM1C directly interacts with the binding domain of DNA Ligase IV, a unique mammalian ligase involved in the final step of ds break repair by NHEJ [60]. Inhibition of the SNM1C/Ligase IV complex formation is of medicinal interest, because decreasing the efficiency of NHEJ repair in tumors might synergize with chemotherapeutic agents and radiotherapy [60]. SNM1C may also have a role in cell cycle regulation via direct interaction with the Cul4A-DDB1 E3 ubiquitin ligase complex, which is required for the degradation of the cyclin-dependent kinase protein inhibitor p27 during the transition from G_1_ to S phase [61].

### ELAC 1 and 2 Proteins (ELAC1/2)

ELAC1/2 are nucleases that mediate 3′-tRNA processing; both belong to the RNAse Z family and contain two active site zinc ions [62]. Human ELAC1 is significantly smaller than ELAC2 (363 versus 826 residues), and is similar to the C-terminal MBL domain-containing region of ELAC2; thus, ELAC1 is likely to have evolved as a consequence of gene duplication [63]. ELAC2 displays activity as a 3′-end tRNA-processing nuclease involved in the maturation of mitochondrial (mt) tRNA 64, 65. Mutations in the *ELAC2* gene correlate with accumulation of unprocessed (mt) tRNAs, leading to impaired mitochondrial translation, which is associated with hypertrophic cardiomyopathy, an often lethal inborn pathology [66]. Similar to ELAC2, ELAC1 has 3′-tRNA-processing activity and appears to have endoribonuclease activity on unstructured RNA [67]. Although they have similar biochemical functions, the ELAC proteins have different subcellular localizations. ELAC1 localizes in the cytoplasm, whereas ELAC2 localizes in both the nucleus and mitochondria [68]. The cytoplasmic localization of ELAC1 suggests that it is responsible for degradation of unstructured RNA [68]. By contrast, ELAC2 reportedly interacts with the -tubulin complex in the centrosome during cell division, suggesting a possible role as a cell cycle regulator. Such a role could explain the correlation between mutations in ELAC2 and tumor onset, and other links between polymorphism of *ELAC2* and prostate cancer development 69, 70, 71, 72.

### The Cleavage and Polyadenylation Specificity Factor hMBLf Proteins

#### CPSF73 and CPSF100

CPSF73 and CPSF100 are members of the multicomponent cleavage and polyadenylation specificity factor (CPSF) complex that includes nuclease activities important in RNA processing [73]. The endonuclease activity of CPSF73 is a key factor in transcription termination and in subsequent processing of 3′-end polyadenylated mRNA precursors [74]. Additionally, CPSF73 has 5′-3′ exonuclease activity and is involved in histone 3′-end pre-mRNA processing [75] (Figure 3B). By contrast, CPSF100 does not have RNA processing activity, likely due to a lack of key ‘catalytic’ residues within the second motif (HXHXDH) of its MBLf domain (the motifs were described above) 7, 8. Indeed, a crystal structure of yeast CPSF100 does not reveal active site-bound metal ions [74]. Comparison of crystal structures for human CPSF73 and the yeast CPSF100 reveals highly similar overall folds despite substantially different sequences [74]. Notably, human CPSF100 can exist as a heterodimer with CPSF73; this interaction is proposed to enable the complex to recruit specific pre-mRNAs for processing 73, 76. The precise function, if any, of the apparently catalytically inactive CPSF100 MBL domain is unknown; it is likely that the overall MBL/β-CASP architecture is necessary for the interaction with CPSF73 and perhaps other components of the CPSF complex (including RNA) to form active the multiprotein complexes required for mRNA processing 76, 77.

#### Integrator Complex Subunit 9 (INTS9) and CPSF73L (INTS11)

INTS9 and CPSF73L are hMBLf proteins that are part of the integrator complex, which binds RNA Polymerase II and regulates the expression of target genes. The integrator complex hydrolyzes small nuclear (sn) RNA sequences as part of their transcription termination-linked processing. Recently, the integrator complex was reported to be fundamental in histone mRNA processing 78, 79. After heterodimerization with INTS9, INTS11 manifests the endonuclease activity necessary for the maturation of snRNA by removal of the 3′ ends of pre-snRNA in a manner that resembles the CPSF73–CPSF100 interaction; that is, INTS9 is catalytically inactive and forms a heterodimer with active INTS11 that then shows endonucleolytic activity 80, 81. At present, there is little structural or biochemical information on integrator complexes; such studies will be vital to understanding how snRNA and histone mRNA bind to the integrator complex.

### Structural Studies on hMBLf Nucleases

At present, crystal structures of four of the nuclease subfamily of hMBLf proteins have been reported, for SNM1A, SNM1B, CPSF73, and ELAC1; all of which bind one or two zinc ions in their active site (Figure 3B). Crystallographic analyses show a single zinc ion in the SNM1A active site (PDB ID: 4B87), but two zinc ions in the SNM1B active site (PDB ID: 5AHO), as observed for ELAC1 (PDB: 3ZWF) and CPSF73 (PDB ID: 2I7T) [74]. More detailed biochemical analyses are required to define the biologically relevant metal usages. The CPSF73 and SNM1A/B structures display high overall similarity, in part because they have both MBL and β-CASP domains (Figure 3B); in each case, both domains are required for catalysis, consistent with the similarity in the reactions catalyzed by each (Figure 2B). By contrast, in ELAC1, the αββα core MBLf structure represents the entire ELAC1 protein, consistent with the different reactions catalyzed by ELAC1 [67]. Despite the emerging structural information, the mechanistic details of the hMBLf nucleases remain to be determined. By analogy with the MBLs, an outline mechanism of action for the SNM1 enzymes is shown in Figure 4C: a zinc ion-activated water mediates nucleophilic attack on the phosphate group, which is also activated and/or bound to a zinc ion.

### Group 2 hMBLf Enzymes: Summary

Group 2 hMBLf proteins comprise nine enzymes involved in DNA repair pathways (SNM1 enzymes) and processing of different types of RNA (CPSF enzymes, ELAC1, and ELAC2). Six of these are characterized by the presence of an additional β-CASP domain, which is required for nucleic acid binding (SNM1 enzymes, CPSF73, CPSF73L, and CPSF100) and nuclease catalysis. Crystallographic studies reveal one or two zinc ions at the active sites of SNM1A, SNM1B, CPSF73, and ELAC1, suggesting possible variations in a related nuclease mechanism. Since no detailed mechanistic studies have been carried out for an MBLf nuclease, further work in this field will be of value, including with respect to the development of selective inhibitors; these are of particular interest for the SNM1 enzymes to extend the use of ICLs producing anticancer drugs. Although all the enzymes in this subfamily are nucleases, they have strikingly different selectivities for their nucleic acid substrates; to what extent these arise from direct enzyme–substrate interactions, and/or from interactions within the multicomponent and dynamic complexes in which the enzymes operate in cells, is not resolved.

## Group 3: Other hMBLf Enzymes: NAPE-PLD and CMAH

### N-Acyl-Phosphatidylethanolamine-Hydrolyzing Phospholipase D (NAPE-PLD)

NAPE-PLD is widely expressed, including in the brain [82], and has an important role in the conversion of metabolic lipids into signaling molecules, such as the neurotransmitter *N*-arachidonoylethanolamine (anandamide), which acts on cannabinoid receptors. In the endocannabinoid pathway, NAPE-PLD catalyzes hydrolysis of NAPEs to form anandamide (AEA) and other *N*-acylethanolamines (Figure 2C) 82, 83. Among *N*-acylethanolamines, *N*-palmitoylethanolamine has attracted attention due to its peroxisome proliferator-activated receptor (PPARα)-mediated anti-inflammatory activity [84]; thus, NAPE-PLD could have a role in the anti-inflammatory response. NAPE-PLD is important for fertility because it is a major regulator of AEA in the uterus during the initial stages of pregnancy and high levels of AEA are associated with early pregnancy failure [85]. Recently, targeted deletion of NAPE-PLD in adipocytes has been reported to induce obesity, glucose intolerance, adipose tissue inflammation, and altered lipid metabolism [86]. The results suggest that targeting signaling lipids derived by NAPE-PLD catalysis may be useful for the treatment of obesity and metabolic syndrome.

A human NAPE-PLD structure has been reported (PDB ID: 4QN9) (Figure 3C), which reveals a dimer and a di-zinc active site where residues belonging to the conserved hMBLf motifs coordinate the metals [87]. NAPE-PLD binds two zinc ions, but it is also active with different divalent metal ions, including Mg (II), Ca (II), Co (II), Mn (II), and Ba (II) [83]. Recently, desketoraloxifene analogs have been identified as broad-spectrum inhibitors of different phospholipase D enzymes, including hNAPE-PLD; these findings may help to better investigate the physiological roles of NAPE-PLD and eventually lead to useful clinical applications [88].

### Cytidine Monophospho-N-Acetylneuraminic Acid Hydroxylase

Sialic acids occur at the termini of sugar chains on the surface-exposed portion of plasma membrane proteins and have important roles in ligand–receptor, cell–cell, and cell–pathogen interactions. The most common forms of sialic acid in animals are *N*-acetylneuraminic acid (Neu5Ac) and its hydroxylated form, *N*-glycolylneuraminic acid (Neu5Gc) (Figure 2C). In all mammals except humans, cytidine monophospho-*N*-acetylneuraminic acid hydroxylas*e* (CMAH) catalyzes the addition of an oxygen atom onto the methyl group of the acetyl group of CMP-Neu5Ac to give Neu5Gc [89]. In humans, CMAH is inactivated by deletion of exon 6, which would encode the hydroxylase active site. The relevant mutation occurred more than 0.5 million years ago, before the Neanderthal divergence 90, 91. In humans, Neu5Gc is detected at low levels, likely derived from food intake [89]. The loss of catalytically active CMAH in humans is proposed to be associated with the acquisition of either resistance or susceptibility to pathogens that preferentially recognize Neu5Gc or Neu5Ac 92, 93.

There is emerging evidence that the catalytically inactive human ‘CMAH-like’ protein has biological roles. It is reported to be overproduced on the surface of adult stem cells and to be involved in activation of Wnt signaling [91]. Overexpression of adult stem cell-specific CMAH leads to an increased cellular uptake of exogenous Neu5Gc, suggesting a role for CMAH in Neu5Gc transport [94]. CMAH is also proposed to be involved in glucose metabolism. CMAH^–/–^ knockout mice manifest an obese phenotype with impaired glucose tolerance and pancreatic β cell dysfunction [95]. The absence of functional CMAH in humans is proposed to be involved in our susceptibility to progressive β cell degeneration and, thus, insulin resistance and type 2 diabetes mellitus [95].

From a biochemical perspective, the mechanism by which (nonhuman) CMAH catalyzes hydroxylation of an unactivated acetyl methyl group is interesting but as yet no structure of CMAH has been reported. Similar to ETHE1, CMAH is likely an oxygenase. However, unlike ETHE1, which apparently only requires iron as cofactor, native murine CMAH activity is stimulated by NADH, cytochrome b5, and NADH-cytochrome b5 reductase 96, 97.

### Group 3 hMBLf Enzymes: Summary

NAPE-PLD and CMAH have different biochemical mechanisms, further illustrating the diverse capacities of the MBLf, although the phospholipase mechanism of NAPE-PLD is related to the nuclease activities of the Group 2 enzymes. Both the two Group 3 hMBLf proteins likely have pleiotropic roles in metabolism. From a medicinal perspective, the links between catalytically inactive human CMAH and diabetes, and between NAPE-PLD and obesity/metabolic syndrome, are notable and are stimulating interest in medicinally targeting their respective pathways. In this regard, NAPE-PLD is of particular interest because of its key role in the endocannabinoid pathway, which has multiple medicinal applications, including in regulation of the anti-inflammatory response.

## Concluding Remarks

The bacterial β-lactam antibiotics hydrolyzing MBL enzymes have a clear and, as far as we know, simply executed role in antibiotic resistance; that is, they add water to β-lactam antibiotics, thereby inactivating them. The available evidence suggests that, with appropriate effort, they will be amenable to pharmaceutical inhibition. The biocatalytic roles of other MBLf enzymes range from hydrolysis reactions (e.g., of thioesters, phospholipids, and nucleic acids, but notably not as yet of peptides and/or proteins), to the more exotic redox chemistry of ETHE1 and CMAH. Importantly, the mechanisms of hydrolysis-type MBLf enzymes are likely related to the well-studies β-lactam antibiotics hydrolyzing MBLs. Thus, the relatively small hMBLf structural family comprises a diverse set of 18 metallo-protein domains, many of which are known to have important and emerging biological roles, including in DNA repair and/or resistance to anticancer drugs (hMBLf nucleases SNM1A–C), regulation of expression (CPSF73), detoxification (Glyoxalase II and ETHE1), brain function (NAPE-PLD), and in metabolism (NAPE-PLD, CMAH) (see Outstanding Questions). In several cases, mutations in the genes encoding hMBLf proteins can have profound pathophysiological consequences. Some of the roles of hMBLf proteins are likely pleiotropic and, at least in the cases of the nucleases (in particular RNA-processing enzymes), the enzymes operate in conjunction with other domains and in dynamic multicomponent complexes. These factors will complicate their prosecution as pharmaceutical targets. However, the core domains of hMBLf proteins have conserved folds in their catalytic domains and, at least at a superficial level, as reveled by crystallographic analyses, unexpectedly similar active sites in terms of metal-binding residues. The available structures provide a basis for inhibitor design, and work with prokaryotic MBLs shows that the family is tractable for small-molecule inhibition. Thus, the hMBLf family should be amenable to a template-based small-molecule probe approach for use in dissecting functional aspects of the roles of hMBLf proteins in regulating expression and in DNA repair, as well as pharmaceutical target validation. By analogy to work on the serine β-lactamases, the development of inhibitors of hMBLf enzymes that are involved in resistance to chemotherapeutic agents, such as cisplatin or mitomycin (e.g., SNM1 enzymes), may be of particular medicinal interest.

## Figures and Tables

**Figure I:**
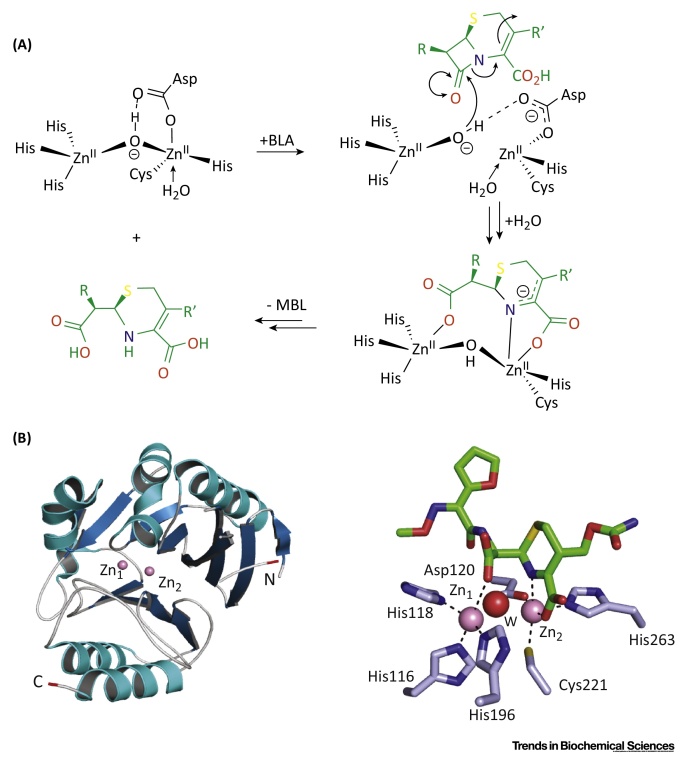
The Reaction Catalyzed by ‘Classical’ Bacterial Metallo β-Lactamases (MBL) Mediates Resistance to β-Lactam Antibiotics. (A) Outline mechanism for the MBL hydrolysis reaction for subclass B1 enzymes exemplified with a cephalosporin antibiotic (in green). (B) View from a crystal structure of NDM-1 β-lactamase (subclass B1) showing the overall fold of a bacterial MBL [Protein Data Bank (PDB) ID: 4RL0] and of its active site in complex with a product derived from cephalosporin hydrolysis (in green). The MBL domain is in blue (β-sheets) and cyan (α-helices). N- and C-terminal residues are in red. Zinc ions are in pink; water molecules are in red.

**Table 1:** Overview of Reported Studies on hMBLf Enzyme Functions, Localizations, and Metal Utilization

hMBLf Proteins (PDB ID)	hMBLf Protein Group	Gene ID; EC; Chromosome Location	Assigned Subcellular Localization	Function/Proposed Function	Reported Metal Content	Linked Diseases	Refs
HAGH (1XM8)	1	3029; 3.1.2.6; 16p13.3	C, M^a^	Glutathione biosynthetic process.	di-Zn (II)	None assigned	31, 113
HAGHL	1	84264; 3.1.2.-; 16p13.3	N/D	N/D	di-Zn (II)^b^	None assigned	
ETHE1 (4CHL)	1	23474; 1.13.11.18; 19q13.31	C, M, N	Glutathione metabolic process. Hydrogen sulfide metabolic process	Fe (II)	Ethylmalonic encephalopathy (EE)	14, 34, 40, 44
LACTB2 (4AD9)	1	51110; 3.-.-.-; 8q13.3	M (by Compara), C (by PSORT II)	N/D	di-Zn (II)^b^	None assigned	
MBLAC1/2	1	255374/153364; 3.-.-.-; 7q22.1/5q14.3	N/D	N/D	di-Zn (II)^b^	None assigned	
PNKD	1	25953; 3.-.-.-; 2q35	I 1: ME; I 2: C (http://www.uniprot.org/locations/SL-0086)	N/D	Zn (II)	Dystonia type 8 (DYT8)	47, 54
SNM1A (4B87)	2	9937; N/A; 10q25.1	N	Cell cycle, DNA damage/ repair	Zn (II)	Mutations: impaired nuclear focus formation, reduced interaction with PIAS and increased sensitivity to cisplatin	15, 61, 62
SNM1B (5AHO)	2	64858; 3.1.-.-; 1p13.2	CH, C, CY, N; T	DNA damage/repair	di-Zn (II)^b^	Hoyeraal–Hreidarsson Syndrome	61, 114, 116
SNM1C	2	64421; 3.1.-.-; 10p13	N	Adaptive immunity; DNA damage, recombination, repair	N/D	Severe combined immunodeficiency autosomal recessive T cell-negative/B cell-negative/NK cell-positive with sensitivity to ionizing radiation (RSSCID), Severe combined immunodeficiency Athabaskan type (SCIDA), Omenn syndrome (OS)	71, 115, 117, 118
ELAC1 (3ZWF)	2	55520; 3.1.26.11; 18q21	N	tRNA processing	di-Zn (II)^b^	None assigned	[82]
ELAC2	2	60528; 3.1.26.11; 17p11.2	M, N	tRNA processing	di-Zn (II)	Prostate cancer, hereditary, 2 (HPC2)	76, 79, 84
CPSF73 (2I7T)	2	51692; 3.1.27.-; 2p25.1	N	mRNA processing	di-Zn (II)	None assigned	88, 89
CPSF73L	2	54973; 3.1.27.-; 1p36.33	N, C	pre-snRNA processing	N/D	None assigned	[94]
CPSF100	2	53981; N/A; 14q31.1	N	mRNA processing	c	None assigned	[90]
INTS9	2	55756; N/A; 8p21.1	N/D	snRNA processing	N/D	None assigned	
NAPE-PLD (4QN9)	3	222236; 3.1.4.54; 7q22.1	ME	Lipid/Phospholipid degradation, metabolism	di-Zn (II)	None assigned	97, 101
CMAHP	3	8418; N/A; 6p21.32	C (http://www.ebi.ac.uk/QuickGO/GTerm?id=GO:0005737)	N/A	d	N/A	[108]

a Abbreviations: C, cytoplasm; CH, Chromosome; CY, cytoskeleton; M, mitochondria; ME, membrane; N, nucleus; PDB, protein data bank; snRNA, small nuclear RNA; T, telomere; N/D, not determined.

b Proposed di-Zn (II) ions observed in the active site inferred by sequence similarity with reported MBL crystal structures.

c Likely a catalytically inactive hMBLf (no metal ion binding observed in the modified ‘active sites’, by crystallographic analyses of the yeast CPSF100 ortholog).

d No metal due to the loss of MBL domain in the assigned pseudogene.

## Box 1 β-Lactam Antibiotic Resistance and β-Lactam Antibiotic Hydrolyzing MBLs

Beginning with the penicillins, successive generations of classes of β-lactam antibiotic, including the cephalosporins and carbapenems, were developed. However, all β-lactam antibiotic classes are vulnerable to resistance mechanisms, including efflux pumps, target mutations, and, probably most importantly to date, their inactivation by β-lactamases, which hydrolyze them into inactive β-amino acids 98, 99, 100, 101. In mechanistic terms, the β-lactamases are divided into those using a nucleophilic serine residue (serine β-lactamases, SBLs; classes A, penicillinases; C, cephalosporinases; and D, oxacillinases) and those using zinc ions (MBLs; class B) in catalysis 1, 2.

Pioneering work led to the discovery of the clinically useful β-lactamase inhibitors clavulanic acid, sulbactam, and tazobactam [102]. Remarkably, these compounds are themselves β-lactams; although they are insufficiently active to be used as antibiotics, in combination therapies they are effective at protecting for example, penicillins from penicillinase-mediated hydrolysis. A new β-lactamase inhibitor, avibactam, is not a β-lactam but does have a reactive carbonyl group; it has been recently approved for use in combination therapies with penicillinases as an inhibitor of class A, C, and some D β-lactamases [4]. The MBLs are concerning because they catalyze the hydrolysis of almost all β-lactam antibiotics and serine β-lactamase inhibitors.

The classical prokaryotic MBLs catalyze β-lactam hydrolysis via a mechanism involving zinc-mediated β-lactam and water activation (Figure IA). They can use either one or two active site-bound zinc ions, although the latter is more common; in isolated form, they can also use transition metal ions other than zinc [103]. MBLs are classified into three subfamilies (B1, B2, and B3 MBLs) based on substrate selectivity and/or sequence/structure-based homologies [8]; all have the characteristic αββα core fold, as first revealed in a MBL II structure from *Bacillus cereus* in 1995 [5]. Distinctive active site elements, including four highly, but not universally, conserved zinc ion-binding motifs occur in the MBLs: (i) H_116_XH_118_XD_120_ (an additional residue His_121_ is only present in subclass B3); (ii) H_196_; (iii) C_221_; and (iv) H_263_ (Figure IB). Note that wherever possible, we use the MBL ‘BBL’ numbering system [104]. The structural work revealed that the MBLf is widespread. To date, >34 000 predicted MBLf proteins have been identified 3, 9, 105, 106; while of these many are ‘classical’ antibiotic resistance MBLs (<1000), many are not 9, 103, 107.

Although gaps remain in our knowledge, a combination of crystallographic, modeling, and solution studies using kinetics and spectroscopic methods is enabling a detailed mechanistic understanding of the prokaryotic MBLs. The zinc ions have central roles in catalysis, not only in activating both the ‘hydrolytic’ water and the β-lactam carbonyl, but also in substrate binding, including via the carboxylate present in most β-lactam antibiotics (Figure IA). Notably, the β-lactam antibiotic aztreonam does not contain such a carboxylate and is not an MBL substrate [108], illustrating that is possible to obtain selectivity in binding to MBLs, even with related compounds. Various MBL inhibitors have been reported, principally in the academic literature, and some patents have been filed 109, 110. Perhaps the best-characterized MBL inhibitors are thiols, which displace the ‘hydrolytic’ water that bridges the two zinc ions that are found in clinically relevant MBLs [18]. As yet, no MBL inhibitors have progressed to the clinic; however, the available evidence suggests that they will be viable targets, at least in term of achieving efficacy.
